# Supplementary material for: Are carbon emissions trading and green financial instruments synergistic? -Comprehensive quantitative research based on content analysis
Source: PLoS One. 2024 Mar 7;19(3):e0298601. doi: 10.1371/journal.pone.0298601 (PMC10919601; doi:10.1371/journal.pone.0298601)
Supplement: S1 Data — (ZIP) [file pone.0298601.s001.zip › Data/地级市Z_F工作报告.zip!/╕╩╦α/╛╞╚¬╩╨/└┤╫╘╣1⁄2╓┌║┼í╛┬φ┐╦╩2╛▌═°í┐_2016.docx]

各位代表:

　　现在,我代表酒泉市人民政府向大会作工作报告,请予审议,并请各位政协委员和其他列席人员提出意见。

　　2015年工作回顾

　　2015年,在省委、省政府和市委的坚强领导下,我们认真贯彻党的十八大和十八届三中、四中、五中全会以及习近平总书记系列重要讲话精神,按照市委“五做五加强”总体思路,突出固定资产投资、精准扶贫、党风廉政建设三项重点,深入实施“3341”项目工程,实现了经济社会持续稳步发展。预计,全市完成生产总值560亿元,增长6%；固定资产投资1124亿元,增长12%；社会消费品零售总额182.2亿元,增长12%；财政总收入100.6亿元,增长7.4%,其中公共财政预算收入33.4亿元,增长5.4%；财政总支出123亿元,增长4.3%；城镇居民人均可支配收入26870元,农民人均纯收入13478元,分别增长9%和11%。政府承诺的资助高校毕业生见习就业、学校改薄工程、保障房建设、安全饮水等10件惠民实事基本完成。一年来,我们的主要工作成效是:

　　1、固定资产投资止滑稳增。坚持把抓项目、扩投资作为稳增长的重要抓手,强化项目建设调度,靠实投资目标任务,加快立项备案和环评安评节奏,开工建设重大项目2821个,其中亿元以上项目245个,争取国拨省扶投资41.4亿元,增长9.4%,扭转了上半年投资下滑的被动局面。敦格铁路敦煌至肃北段建成通车,京新高速白明段、柳敦高速开工建设,百立通用机场完成跑道基础工程,肃航一级公路、敦当高速、酒额铁路、敦煌客运专线等前期工作进展顺利。灌区续建配套、病险水闸加固、中小河流治理、湿地保护等水利项目完成年度任务,敦煌水资源合理利用与生态保护项目有序推进,“引哈济党”工程项目建议书获批。超前谋划对接“十三五”规划,论证储备“六个一批”重大项目48个,获准进入国家和省上PPP项目23个。内引外联加大招商力度,开工建设招商项目1073个,引进到位资金1098亿元,增长20%,一批新能源补链项目和资源综合利用项目落地建设,产业培育发展的协调性明显提高。

　　2、改革开放迈出新的步伐。认真落实民间投资和非公经济市场准入激励政策,全面实施商事制度改革,释放市场潜能,激发创新活力,新增各类市场主体7970户,实现非公经济增加值289亿元,增长8%。深化投融资体制改革,省股权交易中心酒泉运营中心成立,敦煌农商银行挂牌,招商证券酒泉营业部即将开业,银河证券入驻筹建,187家企业在“新三板”和省股权交易中心挂牌,直接融资8.5亿元,增长62.9%。预算管理制度改革深入推进,国库集中支付率达97%,清理盘活存量资金10.2亿元,统筹用于精准扶贫、项目建设、民生工程、政府债务偿还等方面,财政预决算和“三公”经费使用全部向社会公开,公车改革方案上报待批。农村土地承包经营权和农业设施产权确权颁证有序推进,疏勒河流域水权改革试点全面开展,农村电网“两改一同价”改革完成。行政管理体制改革和事业单位改革稳步推进,市区城市建设、土地管理体制进一步理顺,酒泉经开区三定方案审定批复,县市区住房公积金管理机构和酒泉市中医院管理体制调整上划。积极推进对外经济合作,在霍尔果斯口岸成功举办“酒泉中亚特色商品展览会”,引进外商企业40户,引进外资7.7亿美元,实现进出口总额5亿元,增长7%。

　　3、产业结构调整稳步推进。主动适应发展新常态,加快改造传统产业,着力培育接续产业,新兴产业和服务业快速发展,三次产业结构调整为13.6:38:48.4。认真落实强农惠农政策,大力扶持高效设施农业,调减压缩低产低效作物,新建日光温室5013亩、钢架大棚1.5万亩,定植特色林果7.5万亩,新增3000元以上高效田10万亩,新增肉牛2万头、肉羊60万只,肉羊饲养量达到750万只。全市新建产业化龙头项目15个,创建农民专业合作示范社84个,认证农产品知名品牌5个,落实“三品一标”面积126万亩,占食用农产品生产面积的67.4%,农产品抽检合格率达到99.8%。积极应对经济下行压力,制定出台帮扶企业20条措施,着力解决企业生产经营困难,风电二期首批300万千瓦项目建成,二期500万千瓦项目启动,1350兆瓦光伏指标下达,新增光电装机275兆瓦,全市年发电量达到124.5亿千瓦时,阿克塞50兆瓦、敦煌10兆瓦光热发电项目开工建设,玉门科陆风光储输实验示范项目填补全省空白,金塔、玉门、瓜州、敦煌入选全国新能源产业百强县。酒泉至湖南800千伏特高压直流输电工程及配套750千伏变电站开工建设,金塔、玉门、阿克塞330千伏输变电工程即将并网运行。万象建材等103个资源综合利用项目建成,完成节能技术改造项目7个,认定甘肃“专精特新”企业4户,培育发展规上企业20户,工业增加值增长3%。实施商贸流通重点项目98个,改扩建商贸中心和特色商业街16个,东方明都、玉门时代购物广场等5家商业广场开业,肃北县仓储物流中心建成投用,巨龙物流港被确定为省级农产品交易市场。敦煌机场国际航空口岸获批,新增国内直达航线5条,“玄奘之路—瓜州号”旅游动车冠名开通,柳沟物流园搬迁工程完成。开工建设旅游、文化、体育产业项目167个,新增“历史再现”博物馆15个,敦煌市入列首批国家级旅游业改革创新先行区,敦煌地质公园成功申报为世界地质公园,敦煌盛典实景演出向游客开放,瓜州锁阳城、草圣故里和金塔沙漠胡杨林景区升格为4A级,举办丝绸之路(敦煌)国际文化博览会准备工作会、第四届国际文化产业大会暨第八届甘肃省文博会、国际戈壁超级马拉松等41项节会和户外赛事系列活动,旅游接待人数和旅游收入分别增长25%和26%。科技服务、信息咨询、健康服务、“互联网 ”等新业态不断涌现,巨龙、聚馨等电子商务基地建成,实现电商交易额48亿元,增长35%。

　　4、城乡发展面貌持续改善。酒泉市新型城镇化规划、历史文化名城保护规划、城市风貌规划、敦煌和玉门“多规合一”规划等专项规划编制完成。北大河生态治理及河道景观、生态绿地建设工程完成,丝路公园、体育公园、滨河公园向市民开放,新城区二期“四纵五横”道路和滨河南路建成,第二水厂迁址扩建和第二污水处理厂建设工程完成年度任务。

　　老城区6条道路和16个楼院小区环境改造完成,新增供热面积130万平方米、天然气用户2.4万户,开工建设公益性蔬菜市场3个。市容市貌、交通秩序和环境整治深入推进,新增城区绿化面积102万平方米,玉门市创建为国家园林城市,敦煌市创建为国家卫生城市。扎实推进敦煌市国家级,玉门市和航天镇、总寨镇省级新型城镇化试点,新改建农村公路776公里,新增农村安全用水居民1.2万人,新续建楼居式社区化中心村16个,改造农村危旧房2050户,建成美丽乡村示范点34个,城镇化率比上年提高1.5个百分点。城乡生态环境持续改善,完成人工造林11.3万亩,封滩育林10万亩,义务植树471万株；实施污染治理减排项目61个,万元GDP能耗下降3.04%,单位工业增加值用水量下降7.5%,市区空气质量优良天数达到280天以上,环境约束性指标全面完成。

　　5、精准扶贫脱贫成效显著。深入领会习近平总书记扶贫开发战略思想,认真落实省、市委精准扶贫决策部署,按照精准扶贫“四个六”的要求,明确各级干部包抓责任,结合落实省上“1 17”工作方案,对接出台84条配套措施,推行划片包抓、蹲点帮扶、按期脱贫责任考核,实现了精准扶贫全覆盖、精准脱贫见实效。全市共争取落实帮扶资金5亿多元,对接实施扶贫项目76个,帮助贫困村、贫困户办实事1795件,硬化村组道路112公里,改造危旧住房1768户,建设农田林网4260亩,衬砌干支渠308公里,改造中低产田2.5万亩,培训输转劳务2.6万人。着力培育扶贫增收产业,新建日光温室和钢架大棚1024座,新增枸杞、甘草等高效田3万亩,建成暖棚圈舍2079座,户均肉羊饲养量达到35只,移民人均纯收入增长26.4%,预脱贫人口3.2万,超额完成年度目标。

　　6、民生保障水平不断提高。全市投入惠民实事和民生资金31.8亿元,增长12%。大力推进“双创”行动计划,扶持高校毕业生到企业见习就业,新增创业实体6415个,城镇登记失业率控制在2.5%以内。机关事业单位养老保险和城乡居民大病保险启动实施,企业退休人员养老金、城乡低保、五保供养等社保待遇全面提标,平均增幅达10%以上,城乡居民医疗保险和养老保险参保率保持在98%以上。新建城乡社区日间照料中心37个,建成社会化养老机构1个、乡镇养老院2个,市老年服务中心开工建设,肃州、金塔、玉门、敦煌60岁以上老人免费乘坐公交车。保障性住房完成年度建设任务,新建公租房2351套,实施棚改区改造2911户,发放租赁补贴5114户。组织实施科技项目65项,申请专利1159件,酒泉国家农业科技园区获科技部批准。实施校舍安全改薄项目278个,新建校舍16.2万平方米,消除危旧校舍14万平方米,新建市级标准化学校35所,义务教育均衡发展在全省率先整体通过评估考核。医药卫生体制改革取得新进展,县级公立医院综合改革扩大到所有县市,分级诊疗制度全面推开,新农合住院补偿比提高3个百分点,赴基层开展多点执业医师占比达24.7%,乡镇卫生院住院就诊人数提高2.8个百分点,市医院综合项目建成投入使用。中医药事业稳步发展,中医诊疗服务实现县乡村卫生机构全覆盖。市博物馆布展完成并免费开放,市体育馆开工建设,21个文化服务项目、100个数字文化长廊服务点、6个县市数字影院建成投用,新建“图书漂流”服务点64个、乡村舞台115个,承办国家和省级体育赛事9项,举办全市体育赛事35项。食品药品监管体系进一步健全,安全生产四项控制指标全面下降。鼠疫防控措施全面落实,实现人间鼠疫“零发生”目标。民族团结进步创建活动持续开展,双拥模范城建设取得新的成就。“六五”普法全面完成,公民法律意识普遍提高,瓜州县荣获全国法治县创建活动先进单位。深入推进“平安酒泉”建设,健全信访、维稳、综治、民调“四位一体”工作机制,信访案件妥善有效处置,社会治安形势总体平稳,群众对社会治安满意度达98%。统计、人防、边防、地震、气象、妇女、儿童、台侨、档案、史志、外事等工作取得新的成绩。

　　在狠抓经济社会发展各项工作的同时,我们加强学习型、法治型、服务型、廉洁型政府建设,扎实开展“三严三实”专题教育,严格执行中央八项规定和省市委“双十条”规定,大力推进政府职能转变和管理创新,坚决整治行政不作为和庸懒散慢现象,从严控制部门预算,削减压缩一般性支出,“三公”经费下降26%,节约资金2204万元。深化行政审批制度改革,取消合并下放审批事项46项,市、县两级政府“三张清单一张网”建设完成,政府部门权责事项、专项资金管理清单、便民服务事项等政务信息全部公开发布,省市县三级政务服务平台联网运行,规范性文件备案审查率达100%。完善公共资源交易流程,交易目录实现全覆盖,我市被确定为全省远程异地评标试点城市。坚持政府决策集体讨论决定,自觉接受人大法律监督、工作监督和政协民主监督,邀请人大代表、政协委员列席政府常务会,办理人大代表意见建议和政协委员提案372件,办复率达100%。认真落实省委“3783”和市委“3873”党风廉政建设主体责任,持续开展各项规定执行、建设工程招投标、政府采购、财政资金使用等领域专项检查,对21名市管领导干部、24项政策执行、6项重大民生资金、25个政府性投资项目和112个市直部门单位进行了审计,对主体责任落实不到位的13个单位16名领导干部进行了问责,对违纪违规问题进行了严肃处理。

　　各位代表,一年来,尽管经济形势复杂严峻,但我们齐心协力,迎难而上,取得了转型发展的良好业绩,完成了年初确定的目标任务。这些成绩来之不易,是省委、省政府亲切关怀的结果,是市委科学决策、正确领导的结果,是市人大、市政协和社会各界支持监督的结果。在此,我代表市人民政府,向全市百万各族人民,向拼搏奋战在各行各业的建设者,向各民主党派、工商联、人民团体、无党派人士、离退休老同志,向驻酒解放军指战员、武警官兵、公安干警,向中央、省属驻酒单位以及所有关心、支持酒泉发展的各界人士表示衷心的感谢和崇高的敬意!

　　在肯定成绩的同时,我们清醒地认识到,全市经济社会发展还存在一些突出矛盾和问题。一是经济下行压力持续加大,资源性行业应对市场风险不足,服务业发展基础薄弱,稳增长、调结构、促转型的任务艰巨。二是项目建设落地制约因素较多,招商引资项目开工率和资金到位率偏低,固定资产投资持续增长动力不足。三是工业经济运行困难,支柱税源贡献度下降,地方财政收入质量不高,财政收支压力较大。四是粮食、果品等农产品价格下跌,农民持续增收面临较大压力,改善偏远乡村和沿山片区基础设施,大幅增加贫困家庭收入的难度较大。五是个别政府部门工作人员大局意识淡薄,担当意识不强,推诿扯皮现象时有发生,等等。这些问题,既是制约我市当前经济社会发展的难点,也是我们做好下一步工作的重点,我们将进一步健全机制,强化措施,认真加以解决。

　　“十二五”主要成就及“十三五”规划纲要说明

　　各位代表,“十二五”是酒泉发展史上极不平凡的五年,面对复杂多变的国内外形势和经济下行压力,在全市人民的共同努力下,我们积极适应发展新常态,圆满完成了“十二五”规划确定的主要目标任务,经济社会发展取得新的巨大成就。

　　——五年来,坚持政策导向抓机遇,把中央和省市委决策付诸酒泉实践,始终按照“五做五加强”总体思路,以项目建设为抓手,开工建设亿元以上重大项目1372个,累计完成固定资产投资4178亿元,是“十一五”的3.8倍。瓜星高速、瓜敦高速和兰新高铁、额哈铁路建成通车,全市公路总里程达到1.7万公里,比2010年增加3710公里,路网密度达到963公里/万平方公里,98.5%的乡镇和96.6%的行政村通上了油路,开通国内航线21条,农林水利、交通通信、输电线路、能源管道等基础设施更加完备。经济实力显著增强,全市生产总值年均增长8.5%,第三产业比重提高12.2个百分点,人均GDP突破8000美元,稳步进入中等收入行列。财政总收入连续突破整数关口,跨上百亿元台阶,年均增长14.8%,高于经济增速6.3个百分点,人均公共财政收入3238元,居全省前列。城乡居民收入不断提高,连续五年保持两位数增长。

　　——五年来,坚持做精一产增效益,把发展高效设施农业作为富裕农民的有力支撑,大力实施“365”现代农业发展行动计划,肃州、敦煌被授予国家级现代农业示范区,全市发展高效蔬菜、名优果品、现代制种、特色中药材等优势产业210万亩,建成科技示范园区120个,标准化规模养殖小区982个,农民来自设施农业和规模养殖的收入比重达56%,农业增加值年均增长5.6%。农业规模化经营、产业化发展步伐加快,培育农业新型经营主体2800多个,土地流转占比提高到33%,规模化经营占耕地面积的21%,建成国家级龙头企业4个、省级龙头企业29个,形成保鲜贮藏能力40万吨,农产品加工转换增值率达62%。劳动力输转培训常态化,劳务经济占农民收入的比重达27%。

　　——五年来,坚持做强二产促转型,把发展战略新兴产业作为结构调整的重要突破,制定实施培育壮大首位产业、改造提升传统产业等行动计划,新能源装机容量突破1000万千瓦,酒泉千万千瓦级风电基地、百万千瓦级光电基地按期建成,风光电装备制造形成全产业链生产能力,以超高压为骨干、各等级电压相配套的外送电网实现全覆盖,新能源首位产业占工业增加值的比重达到36%。资源综合利用步伐加快,九大传统产业集群崛起,煤化工、硅材料、建筑建材等6个循环经济百亿元产业链增加值年均增长9.6%。酒泉工业园区升格为国家级经济技术开发区,建成省级工业园区1个,各类园区入驻企业800多户,吸纳了27.1%的城镇就业,创造了近七成的地方税收,对工业增长的贡献率达90%以上。

　　——五年来,坚持做大三产扩规模,把发展现代服务业作为提升产业层次的关键举措,深入开展服务业综合改革试点,建成商品交易市场74个、大型综合性商业设施5个、配送机构17家,新建改造农家店703个,商业网点面积达80万平方米,外贸进出口年均增长6.7%,社会消费品零售总额年均增长15.8%,市场规模比2010年翻了一番。华夏文明传承创新区建设全面推进,丝绸之路(敦煌)国际文化博览会成为国家战略平台,锁阳城、悬泉置、玉门关申遗成功,敦煌文化产业园创建为国家级文化产业示范园区,文化产业增加值占GDP的比重达到3.3%。莫高窟数字展示中心等22个旅游项目建成向游客开放,鸣沙山月牙泉创建为5A级景区,文化、体育、养生与旅游业加速融合,户外运动等业态活力增强,旅游收入年均增长33.7%,达到151亿元,占GDP的比重由9.3%提高到27%。物流、快递、信息、金融、电商等服务业快速发展,第三产业对经济增长的贡献率达到50%。

　　——五年来,坚持做美城市提品位,把建设明星明珠城市作为城镇化发展的目标定位,以酒嘉一体化城市规划纲要和酒泉城市总体规划为引领,坚持新区拓展和老城改造并举,完成城市建设投资280亿元,比“十一五”增加2.2倍,七县市区城市建成区面积达到90平方公里,城镇人均住房面积由24.5平方米增加到27.9平方米,城镇保障性住房供给率达到85%,集中供热实现全覆盖,管道天然气普及率达到59%,绿化覆盖率达到37.6%,市政道路、地下管网、电力通信等城市基础设施明显改善,城市综合承载能力和带动辐射效应增强。城乡一体化步伐加快,新农村建设取得阶段性成效,农村饮水安全普及率由55%提高到98%,砖混结构住房比重由41.8%提高到62%,楼居式社区化中心村占比达到10%,城镇化水平达到56.7%。生态文明建设稳步推进,最严格的水资源管理制度全面建立,森林覆盖率由3%提高到5.5%,单位GDP用水量、单位GDP能耗、污染物排放等约束性指标全面完成,环境质量指数由75.8%提高到85.6%。

　　——五年来,坚持做实民生谋福祉,把提高公共服务和社会保障水平作为政府工作的出发点和落脚点,帮助9500名就业困难人员实现就业,累计选拔6398名高校毕业生到基层和企业就业,应届高校毕业生就业率提高到85%。社会保障体系更加完善,城乡五项社会保险覆盖面保持在90%以上,社保基金收入增长28%,企业退休人员养老金、城乡居民基础养老金、城乡居民医疗保险筹资标准及报销水平分别提高50%、25.7%、142%和80%,城乡低保标准分别增长68.3%和1.2倍,五保供养标准翻了一番。扶贫攻坚成效显著,贫困人口由12.01万人减少到2.65万人,五年减少9.36万人。社会事业全面进步,市级标准化学校创建面达100%,九年义务教育保持全省领先水平,我市被评为全国“两基”工作先进市。研发投入占GDP的比重达2%,科技对经济增长贡献率提高到55%。每千人拥有文化“三馆一站”面积103.6平方米,人均体育场馆面积达1.6平方米,每千人拥有医疗床位5.6张、卫生技术人员5.1人,每千名老人养老床位达到33张,均高于全省平均水平。

　　各位代表,回顾“十二五”发展历程,我们抢抓政策机遇,紧盯发展目标,破解发展难题,经受住了转型阵痛、体制改革、经济下行等多重考验,在探索奋进中坚定了发展信心,在实干攻坚中凝聚了各方力量,在创新实践中提高了执政能力和水平,在应对挑战中取得了丰硕成果。这为我们全面认识新常态、主动适应新常态、积极引领新常态积累了宝贵经验,也为我们全面建成小康社会奠定了坚实基础。

　　“十三五”是全面建成小康社会的决胜阶段,制定和实施好“十三五”规划纲要,明确未来5年发展的指导思想、奋斗目标、主要任务,事关全市经济社会持续健康发展,事关全面建成小康社会大局。市委、市政府高度重视“十三五”规划编制工作,在2014年7月就作了具体安排,成立了专门领导机构,多次召开专题会议,对事关酒泉发展的重大问题进行研究决策,今年10月又集中听取七县(市、区)、经开区和各部门专项规划编制情况,对规划编制工作提出了明确要求。市人大常委会、市政协分别提出了专题建议,为规划编制提供了重要参考。党的十八届五中全会和省委十二届十四次全委会召开之后,市政府认真学习,深入领会,进一步理清了规划编制的思路、重点和举措。按照市委三届十一次全委扩大会议审议通过的《中共酒泉市委关于制定国民经济和社会发展第十三个五年规划的建议》精神,集聚各方智慧起草了“十三五”规划纲要征求意见稿,并在全市范围内广泛征求意见,部分市人大代表、政协委员和社会各界人士提出的许多宝贵意见被采纳。《纲要(草案)》的编制过程,是发扬民主、科学决策的过程,是凝心聚力、共谋发展的过程。经多次征求意见、反复讨论修改形成的《纲要(草案)》,已提请大会审议。下面,我就几个问题作简要说明。

　　一、关于形势判断

　　科学分析面临的环境和形势,是谋划“十三五”规划、抓好“十三五”发展的重要前提。综合判断,世界经济形势依然复杂严峻,大宗商品价格大幅下跌,全球经济在深度调整中曲折复苏,新一轮科技革命和产业变革蓄势待发。我国经济发展进入新常态,增速换挡、结构调整、动力转换的压力增大,但仍处于重要战略机遇期,总体向好的基本面没有改变。我市同全国一样,在新常态下,投资拉动效应和资源带动取向减弱,结构转换升级更加紧迫,环境保护、生态安全等风险累积叠加,但随着国家“一带一路”倡议纵深推进,我市的区位交通、人力资源、产业集群、载体平台等优势更加凸显。特别是广大群众全面建成小康社会的强烈愿望,是“十三五”发展的总体环境特征,也是我们迎接挑战、加快发展的强大动力,为我们转型跨越提供了重要支撑和保障。

　　二、关于预期目标

　　《纲要(草案)》围绕市委《建议》提出的提前全面建成小康社会的总要求,确定了“十三五”经济社会发展的主要预期目标:地区生产总值年均增长7.5%,固定资产投资年均增长12%,社会消费品零售总额年均增长10%,财政总收入年均增长8%,城镇居民人均可支配收入年均增长8%,农村居民人均可支配收入年均增长9%,外贸进出口总额年均增长8%。

　　今后五年,经济保持中高速增长是中央明确的目标要求,根据国家全面建成小康社会“两个翻番”要求测算,2020年我市城乡居民可支配收入和生产总值比2010年翻一番,就是我市城镇居民人均可支配收入、农村居民人均可支配收入和生产总值三项指标从15104元、7234元和405亿元分别达到30208元、14468元和810亿元。由于“十二五”时期我市实现了比较高的增速,“十三五”时期三项指标年均分别增长2.4%、1.4%和6%就可以达到。但省上确定“十三五”三项指标增速分别为7%、9%和7.5%,综合考虑我市发展环境、发展基础和潜在增长因素以及需要达到全面小康、转型升级的目标,我们提出“十三五”时期城镇居民人均可支配收入增长8%,农村居民人均可支配收入增长9%,生产总值增长7.5%是经过积极努力可以实现的。从酒泉基础设施欠账大、内生增长动力弱、国内外经济形势不确定因素比较多的情况分析,投资仍是拉动我市经济增长的主要动力,只有保持12%的投资增长速度,才能支撑7.5%的经济增速。在全省提前两年全面建成小康社会,是基于我市主要经济指标位居全省前列,特别是全面小康综合指数达到85.9%,高于全省10.5个百分点,39项小康监测指标已有15项达到目标值来考虑的。今年我们已经有6个县市区贫困人口发生率降至3%以内,2016年将全部降至3%以内,再经过两年的巩固提高,贫困人口这个短板将彻底解决,在2018年稳定脱贫是完全有把握的。这样的安排,既落实了省委省政府对酒泉的要求,也符合酒泉的实际,体现了经济社会发展实现新跨越的目标,更是顺应了广大群众对幸福美好新生活的热切期盼。

　　三、关于重点任务

　　《纲要(草案)》突出了产业结构转型升级、建设区域创新体系、推进新型城镇化、加快生态文明建设、提升开放型经济水平、完善区域基础设施、加快发展社会事业、强化制度保障8个方面的内容。需要重点说明的是,党中央、国务院决定举办丝绸之路(敦煌)国际文化博览会,这一国家战略平台的建立,为我们开放开发提供了重大发展机遇。着眼开放发展,要充分发挥我市地理区位、历史文化、资源禀赋和产业基础优势,主动融入“一带一路”倡议,充分利用文化博览会、新能源博览会等战略平台和沿边优势,面向丝绸之路沿线国家和相关省份,深化国际文化交流合作和经贸合作,加快酒泉国际港务区建设,着力打造丝绸之路经济带重要节点城市；着眼创新发展,立足资源型产业比重大、科技创新能力弱、产学研结合不紧密的市情特征,实施创新驱动发展战略,加大转方式、调结构的力度,重点围绕战略新兴产业、循环经济产业、特色高效农业和现代服务业持续发力,强化企业的主体作用、政策的引导作用、平台的支撑作用,提高科技对经济增长的贡献率；着眼协调发展,从我市城镇化水平不高、城乡移民三元结构考虑,把完善城镇化发展机制、推进精准扶贫精准脱贫、深化户籍制度改革和基本公共服务均等化作为重点,引导农业人口在城镇落户,贫困人口彻底脱贫；着眼绿色发展,把保护好生态环境作为我们赖以生存和加快发展的必要条件,坚持实施生态立市战略,大力推进生态文明建设,加大生态安全屏障保护力度,加强土地、矿产、水资源、能源节约利用和污染防治,切实改善区域生态环境；着眼共享发展,从解决事关群众切身利益的问题入手,加强义务教育、社会保障、基本医疗和公共卫生、文化体育等基本公共服务,提高供给能力、共享水平和保障覆盖面。通过深化重点领域改革保障发展理念顺利实施。

　　2016年工作安排

　　明年是“十三五”规划的开局之年,我们将按照省委、省政府和市委的决策部署,以马列主义、毛泽东思想、邓小平理论、“三个代表”重要思想和科学发展观为指导,全面贯彻党的十八大、十八届三中、四中、五中全会精神和习近平总书记系列重要讲话精神,紧紧围绕“四个全面”战略布局,按照省委“五个最大”要求,坚持市委“五做五加强”的总体思路和“1234567”工作部署,以提高发展质量和效益为中心,以全面深化改革和实施创新驱动战略为动力,统筹推进经济社会发展各项工作,努力提升富裕文明和谐幸福酒泉建设水平。

　　预期目标是:地区生产总值增长7.5%,固定资产投资增长10%,社会消费品零售总额增长10%,公共财政预算收入增长8%,城镇居民人均可支配收入增长8%,农村居民人均可支配收入增长10%,单位生产总值能耗降低2.5%,主要污染物排放控制在省定目标以内。重点抓好九个方面的工作:

　　一、举全力、攻难点,精心办好首届文博会

　　抢抓党中央、国务院决定中国丝绸之路国际文化博览会在敦煌永久举办的重大历史机遇,把办好首届丝绸之路(敦煌)国际文化博览会作为全市工作的重中之重和首要任务,完善组织协调机构,健全工作推进机制,明确职责分工,全力配合国家相关部委和省上相关部门,加快敦煌国际会展中心、敦煌大剧院施工进度,加快宾馆饭店、旅游景点改造提升,加快敦煌机场扩建、瓜敦高速及莫高窟支线等基础设施配套,确保如期达到会务要求。大力开展安保演练和矛盾纠纷排查调处,全方位、立体式、多角度进行宣传发动和舆论引导,为首届文博会顺利召开创造良好环境。

　　二、强基础、补短板,打好精准扶贫攻坚战

　　把精准扶贫精准脱贫作为主攻方向,突出基础设施建设、增收产业培育、发展能力提升三项重点,健全“三位一体”扶贫机制,强化“六个精准”督查落实,打好瓜州、玉门移民安置区扶贫攻坚战,统筹金塔、肃州插花贫困人口扶贫开发。大力推进土地改良和标准化农田建设,强化“123”产业扶贫,扶持发展设施农业、草食畜牧业和优势特色产业,新建日光温室1000座、钢架大棚1000座,新增养殖小区15个,调引基础母羊2万只,新增优质林果、中药材等特色产业种植面积9万亩。稳步推进新兴产业扶贫,抓好20兆瓦光伏扶贫项目建设,发展电子商务服务站12个。支持移民乡村互助资金社发展,引导金融机构网点向农村延伸,完善金融服务机制,落实精准扶贫专项贷款1.3亿元。开展“雨露计划”和精准扶贫劳动力培训计划,完成技术培训3000人,转移就业1万人,提高贫困群众自我“造血”能力,移民人均纯收入增长20%以上,贫困发生率控制在1%以下,七个县市区全部实现整体脱贫。

　　三、抓项目、扩投资,增强经济发展后劲

　　充分发挥投资的关键作用,加大基础设施、主导产业、高成长性产业、民生保障和社会事业投入,开工建设重点项目1331个,完成固定资产投资1236亿元。准确把握国家投资政策导向,挖掘放大资源、区位、产业优势,抓好项目策划推介,争取更多的项目列入国家和省上投资计划。深入推进“6873”交通突破行动,加快敦格铁路、肃航一级公路、百立通用机场等项目进度,开工建设柳敦高速、敦当高速等工程,抓好酒额铁路、敦煌客运专线等项目前期工作,着力构建互联互通的立体交通网络。围绕主导产业和配套产业链,紧盯优势企业招商引资,增强主导产业集聚度和竞争力。落实招商引资考核奖惩制度,抓好签约项目跟踪落实,招商引资到位资金增长10%以上。完善重大项目合力推进和目标倒逼机制,优化项目建设环境和服务保障,力促项目招得来、留得住、快发展。

　　四、转方式、增效益,加快发展现代农业

　　以服务城市、富裕农民为目标,认真落实“三农”扶持政策,深入推进土地承包经营权确权登记颁证、农业设施、经济林确权颁证等农村产权制度改革,完善农村集体产权权能,加快建立以农村产权为核心的农业投融资体制,引导农业由“生产导向”向“消费导向”转变,提高农业质量效益和市场竞争力,努力走产出高效、产品安全、资源节约、环境友好的农业现代化道路。以酒泉国家级农业科技园区和肃州、敦煌国家级现代农业示范园区为引领,转变发展方式,强化科技支撑,突出龙头带动,推进土地流转和规模经营,大力发展高效瓜菜、现代种业、草牧业、优质林果、特色中药材等优势产业,鼓励支持农产品精深加工,建设一批规模化、标准化种养基地,特色产业面积增长3%以上,牛羊饲养量增长5%以上,农产品加工增值率、农业新品种新技术应用率分别提高2个百分点。加大无公害、绿色、有机农产品地理标志认证申报力度,确保农产品质量抽检合格率达到99%,“三品一标”农产品生产面积提高3个百分点,农业增加值增长5%。

　　五、抓创新、促转型,推动工业优化升级

　　实施工业强基工程,培育发展战略性新兴产业,改造提升传统产业,扶持工业集约集群发展,力争工业增加值增长8%。加快酒泉至湖南800千伏特高压直流输电工程建设,全面开工500万千瓦风电、1350兆瓦光电配套项目,确保2017年与特高压直流输电工程同步运营,建成首个10兆瓦光热示范项目。坚持新能源开发与装备制造互保共建,加快引进风机主轴、齿轮箱等补链项目,首位产业增加值增长10%以上。探索建设电力市场交易平台,支持用电大户与发电企业直接交易,鼓励发展风光电蓄能供热、间歇用能等电能消纳项目。做好酒泉热电联供二期、金泉330千伏升压站等项目前期工作,力促常乐电厂400万千瓦火电项目开工建设。积极推进民用核产业园建设,大力发展循环经济,抓好玉门东镇、瓜州柳沟等煤化工项目建设运营,推进建材、冶金、硅材料等产业改造升级,提高资源综合利用率。推动云计算、物联网等与现代制造业加速融合,扶持发展创新型、科技型企业,抓好重点领域、支柱产业和骨干企业创新联盟建设,培育发展战略性新兴产业骨干企业3户,加速科技成果转化为现实生产力。

　　六、建平台、调结构,持续做大第三产业

　　把发展服务业作为稳增长、调结构的着力重点,发挥区位优势,发展通道经济,全力打造丝绸之路经济带上重要的人文交流平台、商贸物流枢纽、户外运动之都和休闲养生福地,促进发展动力由生产型向服务型转换。加快文化产业基地建设,谋划实施一批文化产业项目,文化产业增加值增长25%以上。以高端百货、批发零售为重点,引进发展大进大出的商贸批发业务,开工建设万达商业广场,抓好巨龙农商网、聚馨飞天网等电商平台,扶持做大巨龙物流港、种子产业园、柳沟物流园等物流园区,带动集聚产业和人气。积极推进西四县旅游资源整合开发,以敦煌国际文化旅游名城建设为核心,着力打造资源共享、联动发展的文化旅游目的地；整合东三县旅游资源,充分利用周边地区自然资源和人文资源,着力打造户外运动等体验旅游集散地,力争旅游接待人数、旅游收入均增长25%以上。加快酒泉中医养生产业园建设,推介引进丝路风情主题酒店等高端服务项目,培育发展养生药膳、中医保健、健康用品研发制造等健康服务业,力争第三产业增加值增长8%。

　　七、重规划、严管理,着力改善城乡面貌

　　坚持规划先行,科学修编酒泉城市总体规划,尽快编制城市地下空间开发利用规划和基础设施等专项规划,做好“多规合一”试点。实施汉唐文化新区、新城区二期、北大河生态治理项目区配套设施建设。推进城市精细管理,开展城市建设和市容市貌综合整治,加快“智慧城市”建设,下大气力解决交通拥堵、停车难等问题。完善社区物业管理办法,健全社区居民委员会、物业服务企业、业主委员会协调机制,探索建立自管委员会、小区物业服务站等多元化管理模式,促进物业管理规范化建设。深化全国文明城市、国家卫生城市和国家园林城市创建,打好大气、水体、土壤污染治理攻坚战,加大施工场地、粉尘排放、秸秆焚烧等污染防治,建成酒泉第二污水处理厂,抓好酒泉第二水厂迁址扩建、酒泉和敦煌垃圾场扩建、敦煌水资源合理利用与生态保护工程。实施好森林生态效益补偿、封滩育林、禁牧休牧等生态修复工程,大力开展绿洲边缘、城镇周边和村庄造林绿化。深入开展美丽乡村示范村、示范组创建活动,打造环境优美的宜居乡村。推进以人为核心的新型城镇化,鼓励在城镇稳定就业和生活的农业转移人口进城落户,城镇化率提高1.5个百分点。

　　八、促改革、添动力,营造创新发展环境

　　充分发挥市场对资源配置的决定性作用,用改革激发市场活力和社会创新动力,加快形成有利于创新、协调、绿色、开放、共享发展的市场环境。大力发展非公经济,放宽民营企业经营范围登记管制,深入推进商事制度改革,落实扶持实体经济各项政策,启动实施科技入园行动,推动大众创业、万众创新,非公经济占GDP比重提高1个百分点。引导民营企业依法进入更多领域,鼓励民间资本公平参与城市基础设施和教育、卫生等社会服务项目建设,增强公共产品供给能力。健全政府预算管理体系,建立跨年度预算平衡机制,完善专项资金管理清单制度。创新政府投融资模式,完善金融服务体系,深化农村金融改革,扩大直接融资规模,强化金融风险防范。全方位扩大对外开放,发挥中国(甘肃)国际新能源产业博览会、酒泉经济技术开发区、敦煌国际文化旅游名城等平台优势,加强与丝绸之路沿线国家和地区的经贸合作,提高酒泉知名度和影响力。完善敦煌国际航空口岸通关设施,做好马鬃山口岸复关争取工作。加快国家级种子外贸转型升级示范基地建设,引导企业发挥产业互补优势,引进来、走出去,发展外向型经济,外贸进出口总额增长7%。

　　九、惠民生、办实事,提高群众幸福指数

　　牢固树立共享发展理念,发展依靠人民,发展成果让人民共享。认真落实就业扶持政策,加强就业技能培训,切实解决就业困难人员和高校毕业生就业,鼓励以创业带就业,城镇登记失业率控制在4%以内。推进建立公平可持续的社会保障制度,抓好机关事业单位养老保险制度改革,完善养老保险转移接续办法,健全医疗保险、工伤保险和生育保险制度。稳步提高企业退休人员、城乡居民基本养老、城镇居民医疗保险补助标准。强化社会救助力度,让身处困境者得到关爱。办好人民满意的教育,加强普惠性幼儿园建设,巩固提高义务教育和高中阶段教育,推进职业教育集团化办学,支持民族教育、特殊教育和继续教育发展。健全公共文化服务体系,继续实施文化惠民和全民健身工程,强化文化遗产保护利用。深化县级公立医院综合改革,全面推行分级诊疗和医师多点执业,开展居民健康卡试点,促进公共卫生服务均等化。继续做好妇女“两癌”普查、救助、康复、培训工作,认真落实两孩生育政策,促进人口均衡发展。推进医疗卫生与健康服务业融合发展,鼓励社会力量兴办居家养老服务,全面完成城乡社区日间照料中心建设任务。深化社会治理创新,健全治安防控网络体系,强化公共安全管理,不断提高人民群众安全感。加强安全生产精细化管理,坚决防止重特大事故发生。实施食品安全战略,强化食品药品质量安全溯源管理。健全应急管理机制,落实公共卫生防控措施,确保无人间鼠疫发生。继续做好统计、人防、边防、地震、气象、民族、宗教、残疾人、台侨、档案、史志、外事、驻外联络经济等工作。

　　切实解决民生问题,集中力量办好十件实事:一是选拔1000名高校毕业生进企业见习就业；二是继续抓好薄弱学校改造工程,新建北苑学校,建成酒泉市第四幼儿园；三是新建、改建农村公路600公里；四是再解决1.59万名移民贫困居民安全饮水问题；五是加大体育基础设施投入,在乡村社区配备室外健身器材和体育设施300台(件)以上；六是在全市建立电子商务中心7个、乡村电商扶贫站(点)100个；七是加大政府调控菜价的比重,在酒泉城区再新建公益性蔬菜市场3个；八是完成棚户区改造4180户,新建公共租赁住房1185套；九是基本建成鼠疫应急物资库及卫生应急指挥中心；十是积极争取扩大全市城市天然气使用覆盖面,肃州区完成天然气入户2万户。

　　全面加强政府自身建设

　　新常态对政府自身建设提出了新的更高的要求。我们要强化学习、认真履职、廉洁自律,促进治理体系和治理能力现代化,努力打造人民满意政府。

　　一是加强理论学习,严守纪律规矩底线。健全落实理论学习制度,认真学习党的路线方针政策,坚定理想信念,严守党的纪律,严格执行中央八项规定和省市委“双十条”规定,深化巩固“三严三实”专题教育成果,坚持“严”字当头,在修身、用权、律己上保持定力,突出“实”字为先,在谋事、创业、做人上履职尽责,确保中央和省、市委重大决策部署全面贯彻落实。加强对国家宏观政策、行业法规政策和产业政策的学习研究,在落实政策中寻找机遇、谋划发展、破解难题,进一步增强推动发展的能力和水平。不断深化市情认识,强化责任担当,切实解放思想,坚持讲程序和重创新并举,从实际出发谋划发展举措,努力提高政府工作水平。

　　二是认真履行职责,强化权力监督约束。坚持法定职责必须为、法无授权不可为,全面履行好法律赋予的各项职能。完善落实调研制度,健全经济形势研判、重大决策部署和重大项目建设推进机制,促进政府工作制度化、规范化和程序化。认真执行公众参与、专家论证、风险评估、合法性审查、集体讨论决定等决策法定程序,完善终身责任追究及责任倒查机制,推行政府法律顾问制度。全面梳理政府权力事项,扩大“三张清单一张网”覆盖面,保障广大群众的知情权、参与权、表达权和监督权。自觉接受人大及其常委会的监督,主动接受政协的民主监督,加强行政监察和审计监督,重视社会和舆论监督,形成常态化监督制度。

　　三是坚持务实高效,着力提升服务能力。创新政府管理方式,完善公共服务供给模式,优化财政支出结构,重点保障基本公共服务。加快推进政府购买社会服务,采取招标、委托等方式,引导社会力量和民间资本参与公共服务项目建设,切实提高政府工作效率和行政效能。深化行政审批制度改革,继续取消和下放行政审批事项,削减前置审批,实行投资项目网上核准,强化并联审批和限时办结制度。加快构建社会信用体系,大力倡导诚信文化。依法加强市场监管,创造公平竞争的市场环境。坚持群众路线,健全深入了解民情、充分反映民意、广泛集中民智的工作机制,着力解决事关群众切身利益的“四风”问题,切实提高政府公信力和执行力。

　　四是严格落实责任,建设清正廉洁政府。全面落实党风廉政建设责任制和主体责任,强化“一岗双责”,落实“一案双查”,健全用制度管人、管事、管权的长效机制。突出问题导向,层层传导压力,认真整改存在的问题,做到严明纪律、严格管理。牢固树立过紧日子的思想,严格落实国务院“约法三章”,持续加大建设工程、国土资源、政府采购、财政资金使用管理、解决群众利益诉求等领域监管治理力度,强化行政问责,坚决查处不作为、乱作为、慢作为和违纪违法案件,努力实现干部清正、政府清廉、政治清明,为经济社会发展营造良好政治生态。

　　各位代表!新常态孕育新希望,新希望开启新征程。让我们在市委的坚强领导下,抢抓机遇,齐心协力,务实重干,攻坚克难,奋力谱写建设富裕文明和谐幸福酒泉新篇章!
